# Assessment of a New Change of Direction Detection Algorithm Based on Inertial Data

**DOI:** 10.3390/s23063095

**Published:** 2023-03-14

**Authors:** Roberto Avilés, Diego Brito Souza, José Pino-Ortega, Julen Castellano

**Affiliations:** 1 Department of Physical Education and Sport, Faculty of Education and Sport, University of the Basque Country (UPV/EHU), 01006 Vitoria-Gasteiz, Spain; roberto_aviles1990@hotmail.com; 2 Department of Physical Education, Faculty of Education and Sport, University State of Londrina, Londrina 86057-970, Brazil; diegobritouel@gmail.com; 3 Department of Physical Activity and Sport, Faculty of Sport Science, University of Murcia, Argentina 19, 30720 Murcia, Spain; josepinoortega@um.es; 4 Research Group GIKAFIT, Department of Physical Education and Sport, Faculty of Education and Sport, University of the Basque Country (UPV/EHU), 01006 Vitoria-Gasteiz, Spain

**Keywords:** validation, reliability, inertial sensors, time-motion

## Abstract

The purpose of this study was to study the validity and reproducibility of an algorithm capable of combining information from Inertial and Magnetic Measurement Units (IMMUs) to detect changes of direction (COD). Five participants wore three devices at the same time to perform five CODs in three different conditions: angle (45°, 90°, 135° and 180°), direction (left and right), and running speed (13 and 18 km/h). For the testing, the combination of different % of smoothing applied to the signal (20%, 30% and 40%) and minimum intensity peak (PmI) for each event (0.8 G, 0.9 G, and 1.0 G) was applied. The values recorded with the sensors were contrasted with observation and coding from video. At 13 km/h, the combination of 30% smoothing and 0.9 G PmI was the one that showed the most accurate values (IMMU1: Cohen’s d (d) = −0.29;%Diff = −4%; IMMU2: d = 0.04 %Diff = 0%, IMMU3: d = −0.27, %Diff = 13%). At 18 km/h, the 40% and 0.9 G combination was the most accurate (IMMU1: d = −0.28; %Diff = −4%; IMMU2 = d = −0.16; %Diff = −1%; IMMU3 = d = −0.26; %Diff = −2%). The results suggest the need to apply specific filters to the algorithm based on speed, in order to accurately detect COD.

## 1. Introduction

Agility is one of the essential components in most team sports. It is defined as the ability to move quickly by changing speed or direction in response to a stimulus [1]. In a sport like soccer, each player can make around 700 turns per game [2], something common before scoring a goal [3]. From a biomechanical point of view, change of direction (COD) requires a deceleration phase consisting of several braking steps, followed by a propulsive phase or push towards the desired direction [4,5,6,7]. For this reason, the eccentric-concentric muscular activity in this type of movement plays a fundamental role, and it is important to know the stress or muscular damage that it can cause to a player [7,8,9]. Efficiency in COD mechanics could be related to the player’s agility as well as being an indicator of favorable performance in elite players [10]. An efficient COD mechanic could reduce the probability of long-term injuries [5,7]. Therefore, analyzing the quality of this type of movement, as well as quantifying it over time, could be very beneficial in improving a player’s performance.

To date, there is little information on COD during soccer. The technique used for this type of analysis is video observation and manual recordings [2,3]. However, this methodology makes it extremely difficult to approach the study of COD in a precise, immediate and sustainable way. GPS (Global Positioning System) devices have been the most widely used monitoring resource in recent years [11]. However, low sampling frequency (5–20 Hz) seems to be a limitation for detecting fast movements when the duration is short [12].

Thanks to the integration of micro-electromechanical sensors (MEMS) devices, such as accelerometers and gyroscopes, it has been possible to delve into movement patterns, with the purpose of studying specific movements in sports [13,14,15,16,17,18,19]. Due to this, it is of vital importance to select sensors that will be used appropriately, according to the motor task selected for the analysis [20]. When 3D orientation is required, magnetic sensors (magnetometer) are integrated together with accelerometers and gyroscopes forming an Inertial and Magnetic Measurement Unit (IMMU) [21,22,23,24]. These sensors are fused with different algorithms, capable of estimating the space-time orientation of a body, with the Kalman Filter being the most commonly used [21,22,23,24]. The different signals obtained from these sensors are affected by different sources of error, such as changes in the physical properties of the MEMS [25], oscillations between the device and the body [26], and ferromagnetic disturbances which can alter the magnitude and direction of the magnetic field vector [27], among others.

The use of inertial sensors for COD analysis seems to have an acceptable validity for identifying the angle of rotation, although there is no consensus on the sampling frequency, number and location of the devices [15]. In the studies reviewed, different sampling frequencies were used, being higher (>140 Hz–1500 Hz) when various segments involved in COD such as the leg, pelvis and trunk were included in the analysis, and lower (around 100 Hz) when the objective was to analyze the movement of the whole body [15]. However, it seems necessary to continue investigating which sampling frequency adjusts best when the movement to be analyzed is a COD.

For all of the above reasons, the objective of this study is to describe the validity and reproducibility of the algorithm configured through the use of several inertial sensors from the combination of different percentages of signal smoothing and the minimum acceleration peak used for the detection of the COD. For this, a designed track was used, performed at two race speeds (13 and 18 km/h) and making turns at different degrees (45°, 90°, 135° and 180°).

## 2. Materials and Methods

### 2.1. Participants

Five non-professional athletes participated in this study on a voluntary basis (age: 30.8 ± 2.1 years, height: 1.83 ± 0.03 m, body weight: 76.3 ± 4.7 kg). All participants were former soccer players, had more than five years’ experience teaching soccer-specific movement skills and had no health problems or musculoskeletal injuries in the six months prior to the test, trying to avoid any biomechanical alteration. All the participants were informed about the procedure, as well as the benefits and possible risks of the study. In addition, the development of the study was in accordance with the Declaration of Helsinki.

### 2.2. Instrument

WIMU ProTM system inertial device (RealTrack Systems, Almería, Spain) was used for the study. The device has four triaxial accelerometers, a triaxial gyroscope and a magnetometer that records movement with a configurable sampling frequency (from 10 to 1000 Hz. In order to be able to select the appropriate sampling frequency, a previous pilot study (not shown in this work) was carried out to compare different recording frequencies (e.g., 10, 15, 20, 100, 500 and 1000 Hz). To do this, a repeated analysis was performed on the same sample, changing the sampling frequency on the WIMU ProTM system software. It was necessary to carry out the test at 1000 Hz (maximum frequency), in order to later be able to reduce it. The results showed that frequencies below 15 Hz overestimated the number of CODs, while from 20 Hz, the number of CODs recorded by the different combinations of the algorithm were very similar, with no differences at 50 Hz. However, it was decided to establish a frequency of 100 Hz, to maintain homogeneity in the selected frequency with previously published works [15].

#### 2.2.1. Algorithm Used and Configuration of Parameters

The algorithm developed by RealTrack Systems (Almería, Spain), identifies the horizontal accelerations detected by the device through inertial sensors. These accelerations are related to changes to which the foot is implanted on the ground or changes of inertia (COI) carried out by the player, always projected on the horizontal plane and classified in the anterior-posterior and medio-lateral axes. The algorithm allows the configuration of some parameters to be modified with the double purpose of having a certain sensitivity to detect COI and, on the other hand, not excessive sensitivity that would cause excessive noise in the information, being impractical on the sports field. The parameters used for configuring the algorithm are:

- Minimum intensity peak (PmI): minimum gravitational acceleration (G) that the movement must reach in order to be recorded.
- % Smoothing: smoothing percentage of the circular mean filter that is applied to the signal before processing it with the algorithm.

#### 2.2.2. Detection of Mediolateral and Anteroposterior Acceleration Orientation

**Figure 1:**
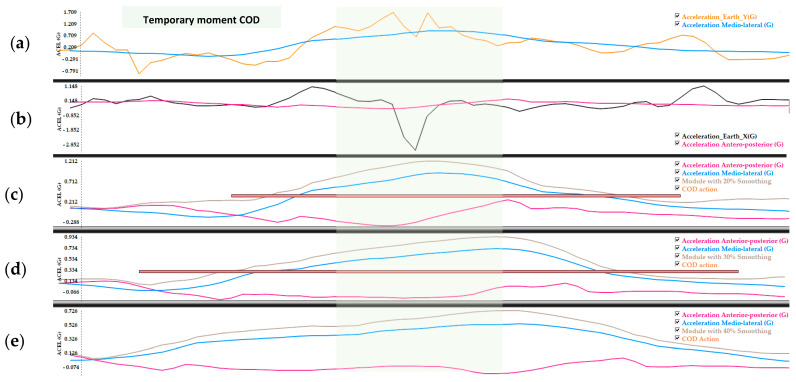
(**a**) Earth Y acceleration signal and its conversion to medio-lateral acceleration. (**b**) Earth X acceleration signal and its conversion to anterior-posterior acceleration. (**c**–**e**), represents the signals unified (module) by the algorithm at 20%, 30%, and 40% Smoothing + Minimum intensity peak (PmI) 0.8 G respectively. The orange bar indicates if the algorithm has detected COD and its duration (length of the bar).

The algorithm used in the COI detection is implemented directly into the SPro “Change of Inertia” module, taking advantage of the modular system of plug-ins (Monitors) available with the SPro software. Each module accesses the data from the sensors/channels generated by each Wimu device. The algorithm gets the information from the data channels EulerX (orientation of the subject in degrees in respect to due north) and Earth X, Y (components of acceleration on the horizontal plane with respect to the earth), through the use of magnetometers. In this way, the EarthX and EarthY values are used to calculate the medio-lateral and antero-posterior acceleration (Figure 1a,b).

#### 2.2.3. % Signal Smoothing and COIs Unification

First, smoothing is applied to the original signal, by means of a circular mean filter whose window depends on the sampling frequency where the sensors are found. To change the coordinate system of the horizontal acceleration vector, the EarthX and EarthY data are re-projected using the EurlerX channel data calculated in the previous step, and the module formed by the new vectors is calculated. Once the module with which the applied horizontal inertia vector has been calculated with respect to the subject, it is possible to detect and count the different COIs. Finally, several COI-type movements are unified into a single COD-type action. This is due to the fact that a single COD can be made up of several COIs, since several steps may be necessary to change the desired direction (Figure 1c–e).

#### 2.2.4. Parameter Settings

The combination of algorithm configuration parameters is shown in Table 1. A total of 18 possible configurations (2 speed from origin × 3 signal smoothings × 3 minimum intensity peaks) were analyzed.

### 2.3. Procedures

**Figure 2:**
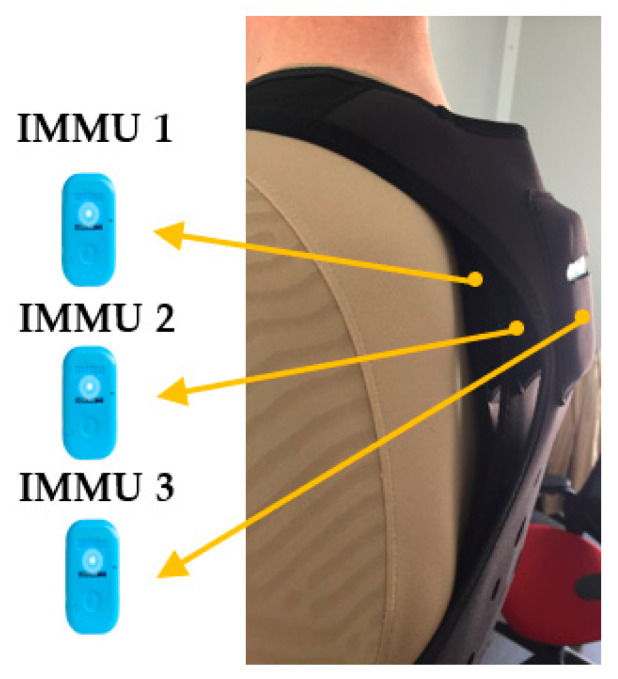
Location of the WIMU devices with the use of the vests.

Before starting the test, the devices were calibrated and synchronized according to the company’s manual. Each participant wore three devices placed in the intrascapular space by means of a bib. The devices were named from inside out: IMMU1 (the device in contact with the back), IMMU2 (device placed between the other two IMMUs), and IMMU3 (the device placed furthest from the body) (Figure 2). The researchers checked that the three devices were perfectly aligned and overlapped one on top of the other prior to performing each repetition. In the perception of the researchers, the devices presented robustly fixed for carrying out the test.

**Figure 3:**
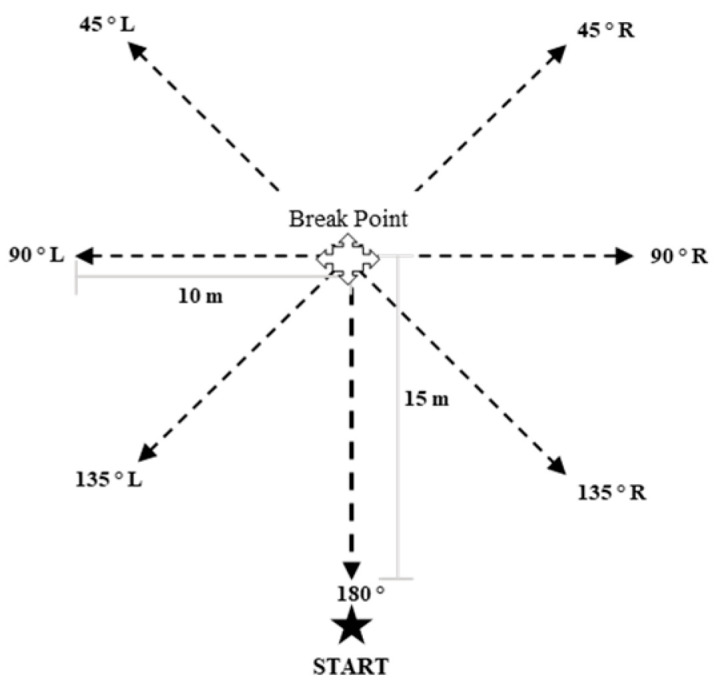
Circuit for testing.

**Figure 4:**
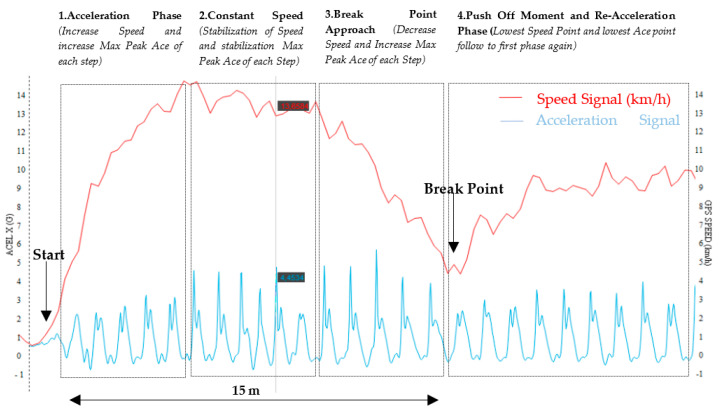
X-Axis Acceleration Channel (blue) and velocity (red) during the course of a COD at 45° at 13 km/h. Different biomechanical phases of a COD.

The test was carried out on an artificial-turf soccer field, with the participants wearing specific soccer boots for that surface. The CODs were repeatedly performed at different angulations and speeds, using a circuit previously used [28] (Figure 3). The participants were instructed to perform a 15 m linear run down a marked line on the soccer field. The researcher, aided by a stopwatch, performed a 3 and 5-s countdown (13 and 18 km/h, respectively) as a reference time in which the breaking point had to be reached in order to do the corresponding COD (Figure 4). In this way, in an attempt to ensure a constant speed in all the repetitions in the test. For a better understanding of the test, the participants were required as a warm-up to do a COD at both speeds in each direction (45°, 90°, 135° and 180°) both to the left and to the right. To carry out the test, each participant performed 5 CODs towards each angle both to the left and to the right at a speed of 13 km/h (Total participant = 40 CODs; Total group = 200 CODs) and 4 CODs at a speed of 18 km/h (Total participant = 32 COD; Total sample = 128 COD). To avoid possible fatigue interference in the dynamics of the test, the participants rested for approximately 60 s between each repetition.

WIMU ProTM system (RealTrack Systems) software was used to analyze and extract the data from the inertial device of each of the recorded repetitions, where a temporary selection of each test repetition was made on the total acceleration channel. Each repetition of the test was subsequently analyzed using the “Change of Inertia” module of SPro. Prior to the statistical analysis, it was corroborated with a video recording using a drone at a height of 15 m above the breaking point (drone: DJI Mavic Pro, DJI, Shenzhen, China) and the Kinovea^®^ software (Kinovea, 0.8.15, http://www.kinovea.org/, accessed on 1 June 2022), each of the repetitions was performed at the selected speed. A total of 15 repetitions were eliminated after observing inconsistency in the approach speed.

### 2.4. Statistical Analysis

An analysis of the normality of data was first performed using the Shapiro-Wilk test. In addition, given the assumptions of normality, a descriptive analysis was performed with mean and standard deviations on the nine proposed combinations. The intraclass correlation coefficient (ICC) and the coefficient of variation (CV) were used to analyze the reproducibility between the different IMMUs and the different combinations at both speeds. For the ICC analyses, an analysis model was used with the average fixed values of evaluators (average fixed raters), a two-way, random effects model. The averaged fixed raters model is a statistical technique that uses a fixed group of evaluators to measure the consistency of measurements. In the case of the different IMMU models, it was considered that they were evaluated by the same group of evaluators, i.e., the IMMU models themselves. Using this model, it was possible to evaluate the agreement levels between the IMMUs models [29]. The coefficient of variation (CV) was used to analyze which of the two modifying parameters of the algorithm (% signal smoothing and PmI) showed greater precision, taking into account the mean and standard deviations of the three IMMUs compared to the movements recorded with video. To analyze the validity of the different combinations in relation to the video, the percentage mean differences were also calculated together with effect size and 95% Confidence Interval, calculated using Cohen’s d, following the classification of [30]: trivial (0.2), small (0.2–0.6), medium (0.6–1.2), long (1.2–2.0), and very long (2.0–4.0). The analysis processes were carried out using Microsoft Excel (Microsoft, Redmond, WA, USA) and IBM SPSS Statistics v.24 (IBM Corporation, Armonk, NY, USA).

## 3. Results

**Figure 5:**
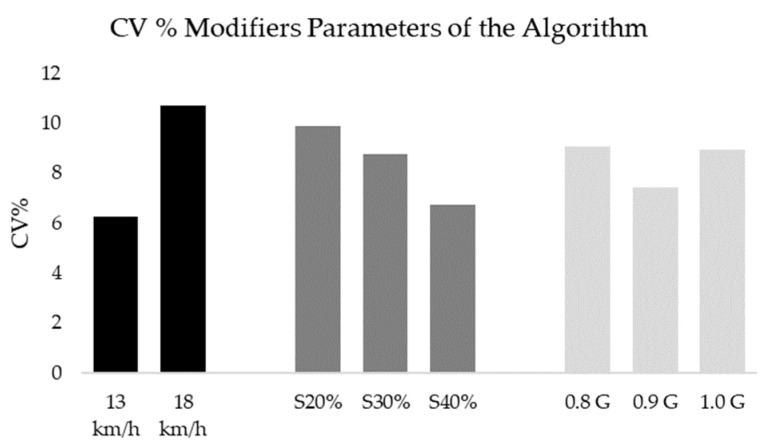
%CV between devices when the combinations were grouped by speed and modifier parameters.

Table 2 shows the mean and standard deviations of video recording and the combinations for the three IMMUs. After a video analysis, participants averaged 38.4 ± 3.6 COD at a speed of 13 km/h and 31.2 ±3.7 COD at a speed of 18 km/h. The ICC test showed a reproducibility level of 0.94 (0.86–0.99) when the algorithm combinations processed the sample at 13 km/h and 0.96 (0.89–0.99) when it was processed over 18 km/h. Figure 5 shows the coefficients of variation between the IMMUs when all the combinations were grouped together with modifier parameters and speed. The results show a lower %CV between devices when all the combinations were pooled at 13 km/h (%CV = 6.5%), a % smoothing of 30% (%CV = 7.9%), and a minimum peak intensity of 0.9 G (%CV = 7.9%).

Table 2 shows the percentage differences, as well as the effect size for all devices and all combinations compared to the video at the speed of 13 km/h. Combinations processed with 20% smoothing showed very large differences (Cohen’s d ≥ 2.8). However, as the minimum intensity peak increased, the % difference between the established COD and the COD registered by the algorithms decreased. The %S20—13 km/h—0.8 G combination was the one that obtained the highest % difference of all the combinations (%Diff ≥100%). However, among the three combinations at 20% smoothing, there was low variability (%CV = 12.8 ± 3.6%). Smoothing at 30% showed more accurate values, showing the combination %S30—13 km/h—0.9 G trivial-small differences in IMMUs 1 and 2 and medium in IMMU3. When the minimum intensity peak was set at 1.0 G, the number of CODs was underestimated (average of the three IMMUs at 1.0 G = 34 ± 1.6 COD; %Diff = −11%). In the same way, when the smoothing was 40%, the values were closer to the number of established CODs. The %S40—13km/h—0.8 G showed small to medium differences with the values being slightly underestimated in the three IMMUs. When 40% smoothing and a minimum intensity peak of 1.0 G were applied, the percentage differences increased negatively in the three IMMUs, being the combination with the lowest values (the mean of the three IMMUs = 28.6 ± 2.3 COD; %Diff = −25.3 ± 5.8%).

At the speed of 18 km/h (Table 2), S20% smoothing showed large differences in the three combinations and the three IMMUs (Cohen’s d ≥ 1.55; %Diff ≥ 104%). As the smoothing and the minimum intensity peak were increased, the values became more precise, showing the combination %S30—18km/h—1.0 G, small and medium differences in IMMUs 1 and 2. For the 18 km/h speed the combination that was most accurate in the three IMMUs was that of %S40—18km/h—0.9 G which showed trivial and small differences in relation to the number of CODs established.

## 4. Discussion

The purpose of this study was to analyze the reproducibility, precision and validity of various configurations of the algorithm processed through RealTrack Systems’ SProTM software using the Wimu Pro device to record CODs with different angulations and velocities. To the authors’ knowledge, this is the first study to present results showing the need to adjust both the smoothing % of the inertial signal and the minimum peak intensity (G) to detect changes in direction. The results obtained show excellent reproducibility, precision and validity of the Wimu Pro device for the detection of changes in direction in a standardized context.

In relation to reproducibility, less variability was found when the run was performed at 13 km/h. This could be related to a decrease in accuracy as speed increases, as has been described for analysis in other GPS variables [12,15]. The IMMU3, a device placed on the outermost part of the subject, was the one that showed the greatest percentage differences in relation to the CODs established at both speeds. This could be due to a slight instability between surfaces, causing slight movements between devices, and worsening when the CODs were performed at 18 km/h [31].

Although the algorithm showed high precision, large differences between the different combinations were found. This seems to explain the need to have to adapt the algorithms to the activity in question. In the present study, both the % signal smoothing and the PmI, were very efficient adjusting the accuracy of the algorithm. When the % signal smoothing and the PmI were set to the lowest settings (%S20 and 0.8 G), the algorithm was not able to remove other movements generated by the subject during the course of the test, resulting in very high percentage differences. This became greater at 18 km/h where speed probably caused greater variability. In contrast, when the parameters were set to maximum (%S40 and 1.0 G), the algorithm underestimated the CODs, perhaps removing too much information, but showing a high potential for bias noise. Similar to the results of this work, high validity and reproducibility were found in the detection of the ball thrown (deliveries) by cricket players and tackles by rugby players, in contrast to manual notes from videos [32,33]. The speed of approach to the moment of COD breakaway seems to be an influential variable in the precision of the algorithm. Thus, the higher the running speed, the more necessary the filter criteria are. In our case, the %S40—0.9 G combination showed the highest accuracy at 18 km/h.

Instead, the speed at 13 km/h, %S30—0.9 G could be enough. This is consistent with previously published data [34], using a similar methodology, where it was clear that as the subject’s acceleration increased in different movements, the percentage differences between the IMMU device and a 3D motion analysis system increased.

Several studies carried out with a GPS signal have shown how modifying the sampling frequency of the device from 5 Hz to 10 Hz seems to increase accuracy when the object of measurement travels distances at high speeds [12,33]. However, that accuracy stopped increasing when it was increased from 10 Hz to 15 Hz [35]. More specifically in inertial sensors, and based on our own analyses, no significant differences were found when the sample was processed above 20 Hz, coinciding with the results of [36], which suggests that for most human movements a 20 Hz sampling is sufficient.

For future research using IMMUs, it seems vitally important to reach a consensus on which filter parameters and thresholds to use for motion detection. Considering the high variability that the different analysis proposals present, it will be difficult to be able to compare results and, consequently, to advance firmly in the understanding of COD.

This work is not exempt from limitations that must be taken into account when interpreting the results. On the one hand, to assess the reproducibility between devices, the three superimposed IMMUs were placed in the intrascapular space of the participants, which could cause slight oscillations between devices that could have affected the results. It would have been interesting to have also tested at other movement speeds such as sub-maximum and maximum. In addition, the fact that the study obtained its results from a standardized test means that the data should be taken with caution when extrapolating them to real activities, such as training or competition matches. In this way, it would be interesting to assess the method in a real-game context, where its natural elements make the player’s movement faster and more unpredictable.

## 5. Conclusions

The novel algorithm provides reproducible, accurate and valid information to detect COD in a standardized test at different angulations and speeds.

## Figures and Tables

**Table 1:** Modifiers parameters of the algorithm.

Start Velocity (km/h)	% Smoothing	Minimum Intensity Peak (PmI)
13 km/h	20%	0.8 G
18 km/h	30%	0.9 G
	40%	1.0 G

**Table 2:** Comparison of the smoothing criterion of COD IMMUs devices between COD video criterion measure at 13 km/h and 18 km/h.

Velocity	%S	G	VIDEO	IMMU1					IMMU2					IMMU3				
			Mean ± SD	Mean ± SD	% Diff	Effect Size (Cohen’s d)	CI 95% (Lower/Upper)	CV (%)	Mean ± SD	% Diff	Effect Size (Cohen’s d)	CI 95% (Lower/Upper)	CV (%)	Mean ± SD	% Diff	Effect Size (Cohen’s d)	CI 95% (Lower/Upper)	CV (%)
**at 13 km/h**	20	0.8	38.4 ± 3.6	78.0 ± 9.3	103	5.62	3.3/7.9	12	83.0 ± 18.6	116	3.33	1.7/4.9	22	91.8 ± 11.7	139	6.16	3.6/8.6	13
		0.9		63.2 ± 6.2	64.5	4.90	2.8/6.9	10	65.5 ± 10.1	71	3.57	1.9/5.2	15	72.3 ± 9.5	88	4.71	2.7/6.7	13
		1.0		54.6 ± 7.1	42	2.88	1.4/4.3	13	56.0 ± 5.2	46	3.90	2.1/5.7	9	59.7 ± 7.3	56	3.71	2.0/5.4	12
	30	0.8		47.2 ± 6.4	23	1.69	0.4/2.9	14	45.0 ± 4.4	17	1.60	0.4/2.8	10	52.0 ± 7.0	35	2.44	1.0/3.8	13
		0.9		36.8 ± 4.4	−4	−0.39	−1.4/0.6	12	38.5 ± 3.7	0	0.02	−1.0/1.0	10	43.5 ± 7.5	13	0.86	−0.2/1.9	17
		1.0		33.8 ± 6.1	−12	−0.84	−1.9/0.2	20	32.8 ± 3.4	−15	−1.61	−2.8/−0.4	10	36.0 ± 5.6	−6	−0.51	−1.5/0.5	16
	40	0.8		36.6 ± 4.4	−5	−0.44	−1.5/0.6	12	35.8 ± 2.2	−7	−0.89	−1.9/0.2	6	37.2 ± 4.4	−3	−0.29	−1.3/0.7	23
		0.9		34.4 ± 7.6	−10	−0.67	−1.7/0.3	22	31.2 ± 1.3	−19	−2.65	−4/−1.2	4	35.0 ± 8.1	−9	−1.69	−2.9/−0.4	23
		1.0		30.4 ± 6.4	−21	−1.54	−2.7/−0.3	21	26.0 ± 3.7	−32	−3.40	−5/−1.7	14	29.5 ± 7.8	−23	−1.40	−2.6/−0.3	26
**at 18 km/h**	20	0.8	31.2 ± 3.7	86.6 ± 26.6	178	2.90	1.4/4.4	31	90.2 ± 35.3	189	2.35	1.0/3.7	39	112 ± 40.8	259	3.00	1.4/4.5	36
		0.9		73.8 ± 26.1	137	2.28	0.9/3.6	35	77.4 ± 28.2	148	2.29	0.9/3.6	36	93.6 ± 34.9	200	2.51	1.1/3.9	37
		1.0		63.6 ± 22.6	104	2.00	0.7/3.2	36	65.6 ± 22.8	110	2.10	0.8/3.4	35	79.2 ± 29.7	154	2.26	0.9/3.6	38
	30	0.8		50.6 ± 13.9	62	1.90	0.6/3.1	27	46.5 ± 11.4	49	1.80	0.5/3.0	24	58.25 ± 16.5	87	2.26	0.9/3.5	28
		0.9		42.6 ± 11.9	37	1.20	0.1/2.4	28	42 ± 10.1	35	1.42	0.2/2.5	24	49.2 ± 10.4	58	2.30	0.9/3.6	21
		1.0		30.6 ± 3.4	−1	−0.16	−1.2/0.8	11	35.6 ± 9.7	14	0.59	−0.4/1.6	27	38.2 ± 1.6	22	2.45	1.0/3.8	4
	40	0.8		41.6 ± 10	33	1.37	0.2/2.5	24	40.8 ± 8.3	31	1.49	0.3/2.6	20	49.4 ± 12.2	58	2.00	0.7/3.2	25
		0.9		29.8 ± 4.0	−4	−0.36	−1.4/0.6	13	30.8 ± 4.1	−1	−0.10	−1.1/0.9	13	30.6 ± 3.0	−2	−0.17	−1.2/0.8	10
		1.0		23.4 ± 7.4	−25	−1.33	−2.4/−0.1	32	29.4 ± 8.9	−6	−0.26	−1.3/0.7	30	29.4 ± 4.9	−6	−0.40	−1.4/0.6	17

## Data Availability

Not applicable.
